# Follow-up for More than 10 Years of Patients with Peritoneal Metastases Treated with Cytoreductive Surgery + Hyperthermic Intraperitoneal Chemotherapy in a Specialized Unit

**DOI:** 10.3390/jcm13010297

**Published:** 2024-01-04

**Authors:** Alba Fernández-Candela, Pedro Bretcha-Boix, Juan Carlos Ruíz Ramírez, Alejandro Paz, Paula Munoz, Miguel A. Ortega, Melchor Álvarez-Mon, José Farré-Alegre

**Affiliations:** 1 Peritoneal Carcinomatosis Unit, General Surgery Department, Hospital Quironsalud Torrevieja, 03184 Torrevieja, Spain; alba.fernandezca@quironsalud.es (A.F.-C.); alejandro.paz@quironsalud.es (A.P.); paula.munozm@quironsalud.es (P.M.); jose.farre@quironsalud.es (J.F.-A.); 2 Pharmacy Department, Hospital Quironsalud Torrevieja, 03184 Torrevieja, Spain; juan.ruizram@quironsalud.es; 3 Department of Medicine and Medical Specialties, Faculty of Medicine and Health Sciences, University of Alcalá, 28801 Alcalá de Henares, Spain; miguel.angel.ortega92@gmail.com; 4 Ramón y Cajal Institute of Sanitary Research, 28034 Madrid, Spain; mademons@gmail.com

**Keywords:** peritoneal metastases, long-term follow-up, overall survival, free-disease survival, cytorreductive surgery, hyperthermic intraperitoneal chemotherapy, peritoneal carcinomatosis

## Abstract

Cytoreductive surgery (CRS) and hyperthermic intraperitoneal chemotherapy (HIPEC) have demonstrated their impact on disease-free survival (DFS) and overall survival (OS) of patients with peritoneal metastases (PM). However, prior literature lacks evidence regarding any follow-up beyond 5 years. In this study, we analyse long-term OS and DFS (more than 10 years of follow-up) of patients undergoing CRS + HIPEC in a specialized unit. We conducted a retrospective study that included only patients who underwent CRS + HIPEC from January 2001 to May 2012. Data collection was conducted by reviewing medical records and telephone calls to patients or relatives. A total of 86 patients were included. The mean PCI was nine (range 0–39) and complete cytoreduction (CC-0) was reached in 80% of patients. Postoperative complications Clavien–Dindo III-IV occurred in 27.9% of patients and the 30-day mortality rate was 2.3%. After 10 years of actual follow-up, OS was 33.7% and DFS was 31.4%. Considering the historical context in which the standard of care for patients with PM was palliation, the results obtained show that CRS + HIPEC was a valid option, with morbimortality comparable to other major abdominal surgeries and encouraging survival results, since, after 10 years of follow-up, almost one-third of patients are still alive and disease-free.

## 1. Introduction

During the last two decades, cytoreductive surgery (CRS) and hyperthermic intraperitoneal chemotherapy (HIPEC) have changed the prognosis of patients with peritoneal metastases (PM). Patients with this condition were considered terminal and showed a median survival rate between 3 and 9 months depending on the primary tumour involved [1,2,3,4]. Nevertheless, in 2003, Verwaal et al. [5] published a randomized trial in which patients with colorectal PM undergoing CRS plus HIPEC showed a median survival rate of 22.6 months versus 12.6 months in patients following standard systemic chemotherapy (*p* = 0.032). These findings were confirmed by Glehen et al. [6] in 2004, who also concluded that complete cytoreductive surgery was the most important prognostic indicator. Similar results were published regarding ovarian cancer [7], gastric cancer [8], small bowel mucinous adenocarcinoma [9] and pseudomyxoma peritonei [10].

Following this favourable data, many institutions implemented this technique around the world, including Spain. Our health center, was one of the nine Spanish centers that started a specialized unit in 2001 [11]. Currently, after 20 years of experience, some centers have published their results [12,13], including all patients that have undergone CRS until now, with a heterogeneous follow-up period ranging from 1 year to 20 years. Only Kim et al. [14] have published results including a homogeneous follow-up period with 88.3% of patients with ten or more years of follow-up. According to that study, only ovarian PM patients were included and they obtained a ten-year survival rate of 19.3% in the CRS group versus 9.1% in the neoadjuvant chemotherapy group (*p* < 0.001).

The aim of our study is to analyse overall survival and disease-free survival rates after 10 or more years of follow-up in patients with PM that underwent CRS and HIPEC in our specialized unit.

## 2. Materials and Methods

### 2.1. Study Population

All patients treated for peritoneal metastases within a peritoneal surface malignancies program at the Quironsalud Torrevieja Hospital from January 2001 to May 2012 were initially considered. Finally, only those who met the selection criteria and had a complete follow-up were included. A retrospective cohort study was conducted from a prospectively gathered database. In May 2022 all patients included were contacted by phone to establish their health status.

### 2.2. Selection Criteria

Patients with acceptable performance status (ECOG < 2), age between 18–70 years old, live expectancy > 12 weeks, adequate haematological count (PNN ≥ 1.5 × 10^9^, platelets ≥ 100 × 10^9^/L) and correct hepatic function (total bilirubin ≤ 1.5, AST (GOT) and ALT (GPT) ≤ 3, alkaline phosphatase ≤ 3) and absence of retroperitoneal lymph node disease, extraperitoneal metastases, intestinal occlusion and serious heart/lung/liver/kidney disease, were included in the study.

Patients with mesenteric retraction in CT images, bladder infiltration, extra abdominal metastasis or unresectable liver metastases, another malignant tumour, multiple intestinal obstructions or active infection, were excluded.

### 2.3. Patient Data

Demographic variables were obtained, including age, sex, carcinomatosis origin and neoadjuvant chemotherapy. Neoadjuvant therapy was guided by tumour origin, therefore, colorectal patients followed the FOLFOX scheme (folinic acid + 5-florouracil + oxaliplatin), gastric patients followed the FLOT scheme (5-florouracil + leucovorin + oxaliplatin + docetaxel) and ovarian patients received carboplatin + taxol. Surgical variables such as peritoneal cancer index (PCI), grade of cytoreduction (CCS) and number of reinterventions, were also gathered. The PCI was categorized as low (1–9), medium (10–19) and high (≥20). Clavien–Dindo classification [15] was followed to grade the 30-day and in-hospital postoperative complications and were subclassified as none (I), minor (II) and major (III–V). Hospital length of stay was also included.

### 2.4. Procedure

After monitoring the patient and administering a balanced general anaesthetic, a medial xipho-pubic laparotomy was performed to carefully examine the abdominal cavity. This allowed us to obtain the tumour load as well as the PCI, following Sugarbaker’s method in which the abdomen is divided into 13 areas (0–12). We took biopsies from every area and cytological samples. The resection of the primary tumour was completed according to oncological criteria (R0 margins and lymphadenectomy), as well as peritonectomies and debulking when carcinomatosis was present, if not, we did not perform extensive systematic peritonectomies. Regarding mesenteric peritoneum metastases, we conducted acceptable small bowel resections in areas of maximum tumour volume and small implants were fulgurated with electrocautery. If no macroscopic implants were left, the cytoreduction was considered complete (CC-0). If residual implants remained, the cytoreduction was considered CC-1 when they were <2.5 mm, CC-2 when they were between 2.5 mm and 2.5 cm, and CC-3 when they were >2.5 cm [16]. The anastomoses were performed after the HIPEC was applied using Sugarbaker’s open coliseum technique. We connected four 36-Fr drains to a continuous closed circuit and placed two intraperitoneal thermal probes to obtain an accurate temperature feedback. An extracorporeal circulation machine (Performer Rand^®^, Modena, Italy) delivered the perfusate at a flow of 500 mL/min and the heat exchanger raised the temperature of the fluid to 48 °C. Once we obtained this temperature, we initiated the drug administration, diluted in 3–5 L of 5% dextrose peritoneal dialysis fluid. The choice of drug depended on the primary tumour, as well as the length of the perfusion. In colorectal and appendicular tumours, we followed Sugarbaker’s protocol and administered 12.5 mg/m^2^ in women and 15 mg/m^2^ in men of mitomycin C for 90 min; after Elias et al. [17] study, we changed protocol to 460 mg/m^2^ of intraperitoneal oxaliplatin during 30 min, with an intravenous bolus of 600 mg of 5-fluoracil 30 min prior to infusion. Originally, for ovarian tumours we would administer Taxol for 60 min, but after Elias et al., we changed the protocol to oxaliplatin or cisplatin + doxorubicin or Taxol for 90 min. In gastric tumours and mesothelioma, we employed oxaliplatin for 30 min. The surgeon distributed the fluid in the cavity periodically during perfusion, and the hemodynamic response of the patient was carefully monitored. The liquid temperature in the abdominal cavity fluctuated between 42° and 43 °C. Subsequently, the liquid was drained and a peritoneal lavage was performed. Twenty-four hours later, early postoperative intraperitoneal chemotherapy (EPIC) was initiated with 650 mg/m^2^ of 5-FU. The dose was kept for 23 h in the peritoneum and administered daily for 5 consecutive days. EPIC was administered until 2008 to all patients as a standard of care during this period.

### 2.5. Follow-up

All patients were seen at the outpatient clinic by oncologists and surgeons every 3 months for the first 2 years, every 6 months until 5 years after the surgery, and once a year thereafter. The follow-up consisted of physical examination and measurement of serum tumour markers on every visit and thoracoabdominal CT scans on alternative visits.

Follow-up after 10 years from the surgery was carried out by reviewing medical records and by phone. Patients were classified as alive and disease-free, alive but with disease, dead due to the disease (PM) or dead due to other causes. The date of relapse and date of death were also gathered. A follow-up was considered complete either through a successful contact by phone with the patient or relatives or knowledge of the date of death of the patient.

### 2.6. Statistical Analysis

The Competing Risk Analysis was used to calculate cumulative incidence of death (CID) and cumulative incidence of recurrence (CIR). CID encompassed from the date of the first CRS + HIPEC until the patient’s death by cancer or until the last follow-up. CIR included from the date of CRS + HIPEC until the first patient’s recurrence or until the last follow-up. Continuous variables were expressed as mean values (range). Categorical data were given as frequencies and proportions. All statistical analyses were conducted by IBM SPSS Statistics (version 27) and R version 4.3.0 (R Core Team, 2023) using package cmprsk (version 2.2-11—Gray B, 2021) for analysis of competing risk.

## 3. Results

### 3.1. Study Population and Surgical Outcomes

During the period between January 2001 and May 2012, many patients were evaluated in our specialized unit, but only 88 met the selection criteria for CRS + HIPEC. Finally, the complete follow-up was achieved in 86 patients, who are included in our sample. Of those 86 patients, twenty-three were men (26.7%) and sixty-three women (73.3%), with an overall mean age of 56 years old. Baseline and surgical characteristics are described in Table 1.

Ovarian cancer was the most common peritoneal metastase origin (44.2%), followed by colorectal cancer (36%) and gastric cancer (10.5%). Mean PCI was 9, ranging from 0 to 39, and complete cytoreduction (CC-0) was achieved in 80% of patients. As far as intraperitoneal chemotherapy drugs are concerned, oxaliplatin was the most used (64.7%), followed by mitomycin C (16.5%) and Taxol (12.9%).

### 3.2. Postoperative Outcomes

Postoperative complications occurred in 53.5% of patients, with 27.9% of major complications (Clavien–Dindo III–VI). The 30-day mortality rate was 2.3% and the mean postoperative hospital stay was 17 days. Details are outlined in Table 2.

### 3.3. Survival Outcomes

**Figure 1:**
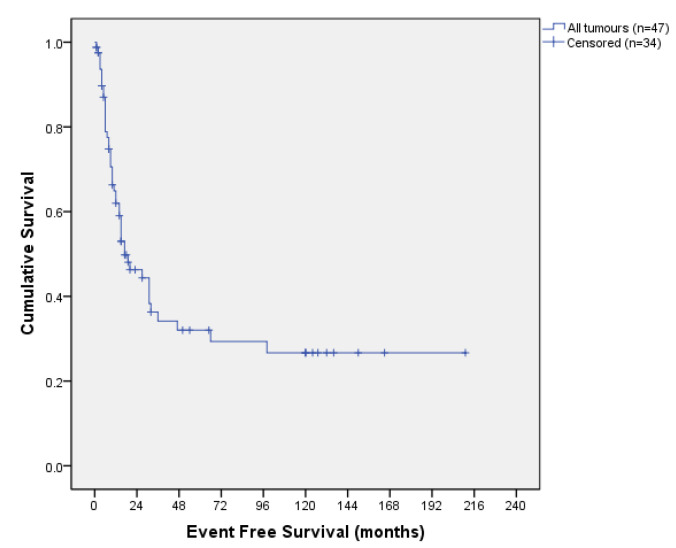
Disease-free survival curve.

The median follow-up period was 66 months. The disease-free survival (DFS) curve is shown in Figure 1. The overall median DFS was 19 months with a 12- and 36- months DFS of 62.8% and 34.6%, respectively.

**Figure 2:**
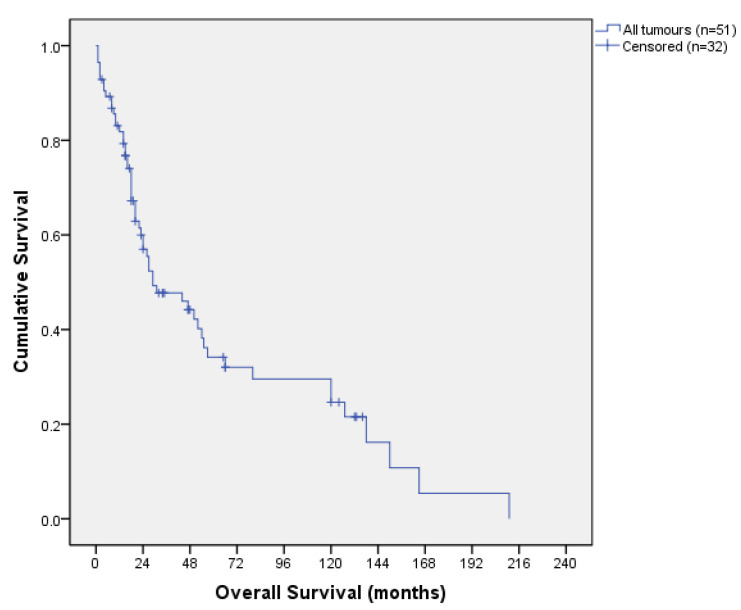
Overall survival curve.

The overall survival (OS) curve is shown in Figure 2. The median OS was 29 months, with a 12- and 36-month OS of 81.8% and 47.7%, respectively. After a 10-year follow-up, OS was 33.7% and DFS was 31.4%. At the time we conducted the survey (May 2022), 27 patients were alive and disease-free.

#### 3.3.1. Survival Outcomes According to Tumour Origin

**Figure 3:**
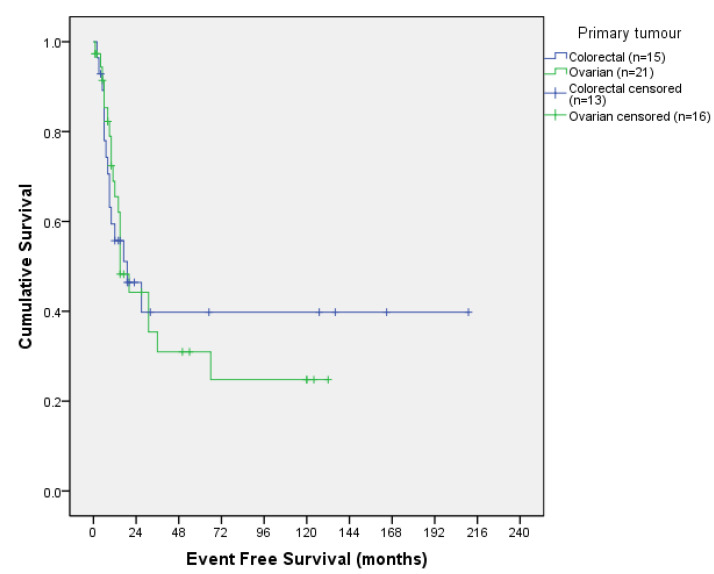
Ovarian and colorectal disease-free survival curve.

**Figure 4:**
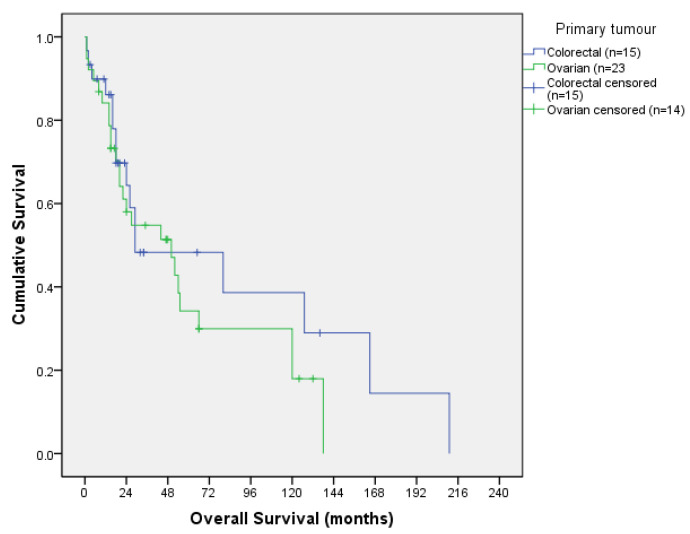
Ovarian and colorectal overall survival curve.

We analysed survival outcomes in the two main tumours of the series, colorectal and ovarian. Curves are shown in Figure 3 and Figure 4.

Colorectal median DFS was 19 months with a 12- and 36-month DFS of 55.7% and 39.8%, respectively; and median OS was 29 months, with a 12- and 36-month OS of 86.1% and 48.3%, respectively.

Ovarian median DFS was 15 months with a 12- and 36- month DFS of 65.5% and 31%, respectively; and median OS was 50 months, with a 12- and 36- month OS of 84.1% and 54.8%, respectively.

#### 3.3.2. Survival Outcomes According to PCI

**Figure 5:**
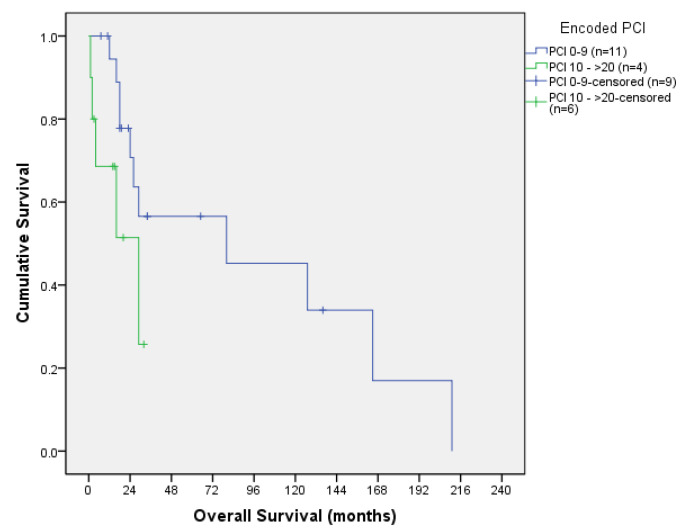
Colorectal overall survival curve according to PCI.

**Figure 6:**
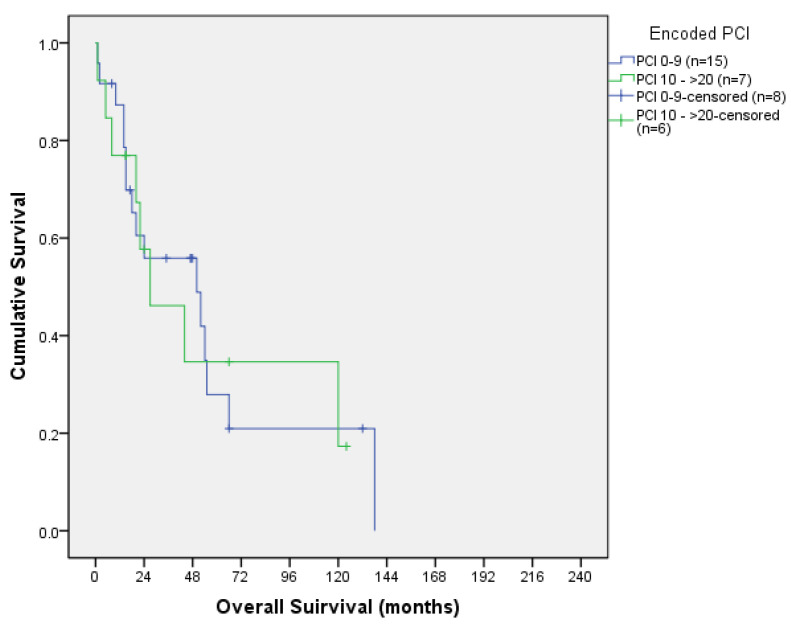
Ovarian overall survival curve according to PCI.

Colorectal and ovarian OS according to PCI are shown in Table 3 and Table 4, respectively, and their corresponding OS curves can be seen in Figure 5 and Figure 6.

When analysing the OS curves, we combined PCI 0 with PCI 1–9 and PCI 10–19 with PCI ≥ 20 to obtain more statistical power, since no differences were found in survival when comparing the four groups separately. Still, we did not reach statistical significance.

#### 3.3.3. Survival Outcomes According to CCS

**Figure 7:**
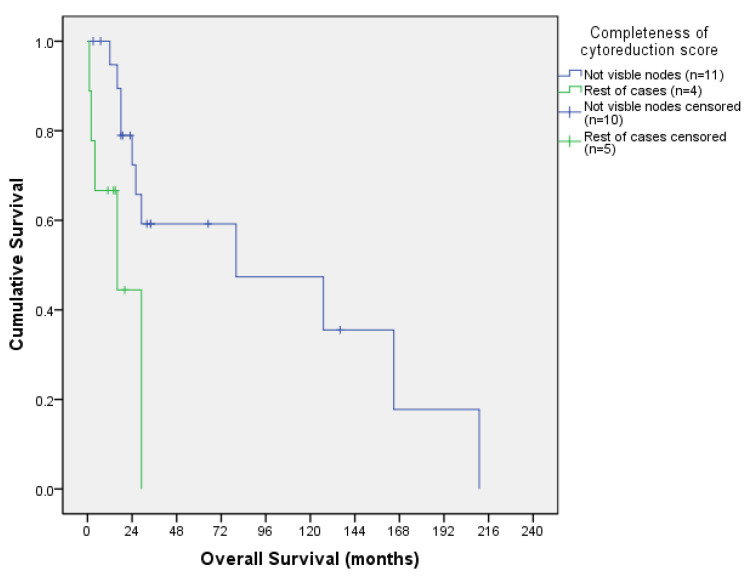
Colorectal overall survival curve according to CCS.

**Figure 8:**
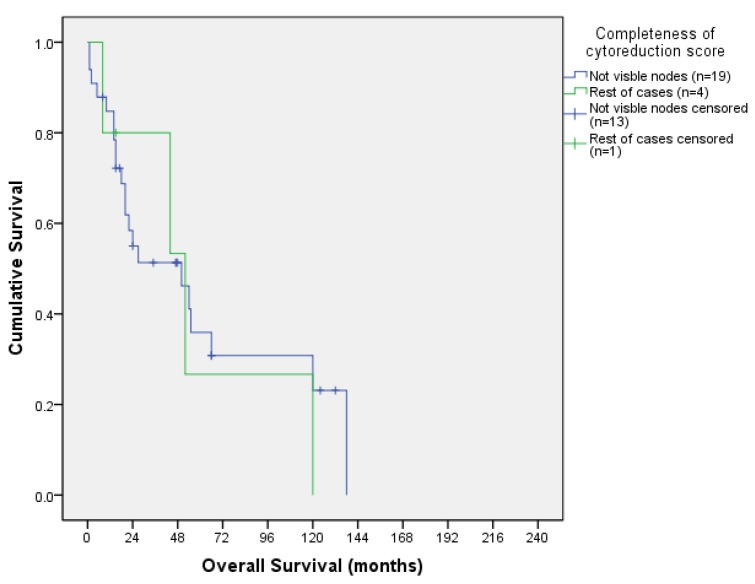
Ovarian overall survival curve according to CCS.

Colorectal OS according to CCS is shown in Table 5 and its corresponding OS curve in Figure 7, while ovarian OS according to CCS is shown in Table 6 and its corresponding OS curve in Figure 8.

When calculating the curves, we combined CCS 0 with CCS 1 and CCS2 with CCS3 to obtain more statistical power. In colorectal patients, the comparison of the two survival curves by Log Rank Test showed a statistically significant difference (X^2^ = 4.411; *p* = 0.036): patients with no visible nods (CCS 0 and CCS 1) had better OS curve than patients with visible nodes (CCS 2 and CCS 3). In ovarian patients, no statistical differences were found between groups.

## 4. Discussion

To our knowledge, this is the first study to be published in which long-term survival in patients with PM undergoing CRS +/− HIPEC is evaluated and all patients included (100%) have 10 or more years of follow-up. We obtained a global overall survival (OS) rate of 33.7% and an ovarian OS of 30%. These results are superior to the results published by Kyang et al. [12] who published an OS rate of 8%, and Kim et al. [14] who published an ovarian OS of 19.3%; but similar to the OS rate published by Ntatsis et al. [13], which was 39%. However, in these studies, the follow-up periods are heterogeneous, and some patients included do not have a 10-year follow-up period. Specifically, in Kim et al. [14] study, 14.7% of patients had less than 10 years of follow up and Kyang et al. [12] and Ntatsis et al. [13] included patients that underwent surgery until 2018 and 2019, respectively.

When analysing our survival results, it is important to remark that patients included in our study had a PCI that ranged from 0 to 39. In the early 2000s, we knew that the successful management of peritoneal metastases relied on several factors such as the presence of comorbidities, the disease stage, the tumour biology or the completeness of cancer excision [18]. In 2004, Glehen et al. [4] identified the limited extent of PCI as a positive prognostic independent factor. Ten years later, in 2014, Cascales et al. [19] published an article in which factors associated with a poor perioperative outcome were analysed and a PCI upper than 12 (OR = 2.942 95%: 1.892–9.594 *p* = 0.044) was an independent factor associated with postoperative morbidity. More recently, in 2022, van Stein et al. [20] concluded that the extent of peritoneal metastases is an independent predictor for completeness of CRS and has independent prognostic value for progression-free survival and overall survival. This evidence is consistent with our results, since we obtained lower OS rates in patients with higher PCI and higher CCS, both in colorectal and ovarian patients, even though, we only reach significance in colorectal patients when comparing CCS, probably due to our small sample. But this proves the impact of tumour burden, represented by PCI, and an adequate cytoreductive surgery, represented by CCS. Moreover, our complete cytoreduction rate (CC-0) of 80% can also explain our OS rate despite our wide PCI range, since incomplete cytoreduction has a negative influence on survival [20,21].

As far as PCI 0 patients are concerned, we should point out that the vast majority were ovarian patients treated with neoadjuvant chemotherapy in which CRS + HIPEC was a consolidation treatment being the last session of chemotherapy; or were high-risk colorectal patients in which a second-look surgery was scheduled to diagnose PM before having analytical or radiological evidence of recurrent disease. Patients with a colorectal tumour with synchronous and localized PM removed, with resected ovarian metastases or with a perforated tumour during first surgery, were considered high risk. In those patients, following the data available at that moment from prospective non-randomized studies, HIPEC with Oxaliplatin for 30 min was administered after a complete exploration of the abdominal cavity. Now, after the Prophylochip trial’s publication in 2020 [22], we know that systematic second-look surgery plus oxaliplatin does not improve DFS compared to standard surveillance, and, as a consequence, now we do not perform second-look surgery in these patients as a standard of care. The rest of the PCI 0 patients were four locally advanced gastric tumours with positive cytology during the first surgery in which a second-look surgery was scheduled after neoadjuvant chemotherapy, and one mesothelioma, which was referred from another center to perform a second-look surgery.

Even though our experience in CRS and HIPEC has evolved and improved over the last two decades, the major morbidity rate (27.9%) and mortality rate (2.3%) obtained, were already consistent with the morbimortality rates published thereafter [23,24], and that was similar to other major abdominal oncological surgeries such as Whipple’s procedure, esophagectomy or hepatectomy [25]. Another point to be considered when discussing morbimortality is the fact that patients included until 2008 underwent early postoperative intraperitoneal chemotherapy (EPIC) after CRS + HIPEC. This procedure required that patients remained in the intensive care unit on postoperative days 1 to 5, receiving chemotherapy through abdominal drains [26]. This technique enabled to deliver chemotherapy at the peritoneal surface to eliminate any residual microscopic tumour cells before the formation of adhesions. Unfortunately, postoperative complications such as fistula were more frequent, and long-term OS was worse than in patients receiving only HIPEC [6,27]. Consequently, EPIC could have impacted negatively in our survival rate and morbidity results, since 83.7% of our patients received EPIC. In fact, we published our results at that time and we obtained a 5-year OS of 30% with a morbidity of 40% [28].

The main limitation of our study lies in its retrospective nature, although no prospective long-term studies have been published addressing OS in PM. Another limitation is the small sample used (less than 100 patients) which was gathered from a single center and includes different origins of peritoneal metastases. Our main strength is the long-term follow-up as well as its consistency since all patients included had 10 or more years of follow-up.

To sum up, considering the historical context in which our specialized unit was founded, where patients with PM only received palliative care and CRS + HIPEC was being implemented around the world by a few centers, we conclude that CRS + HIPEC was a valid option. We obtained a morbimortality rate comparable to other major abdominal surgeries and promising survival rates, since, after 10 years of real follow-up, almost one-third of our patients are still alive and disease-free.

## Figures and Tables

**Table 1:** Baseline and surgical characteristics.

	*n* = 86
Age	56 (24–78)
Sex	
Men	23 (26.7%)
Women	63 (73.3%)
Neoadjuvant therapy	
No	12 (14%)
Yes	74 (86%)
Origin	
Ovarian	38 (44.2%)
Colorectal	31 (36%)
Gastric	9 (10.5%)
Appendicular	5 (5.8%)
Mesothelioma	3 (3.5%)
PCI (mean)	9 (0–39)
PCI	
0	22 (26.2%)
1–9	33 (39.3%)
10–19	15 (17.9%)
≥20	14 (16.7%)
PCI 0	
Ovarian	10 (45%)
Colorectal	7 (31.81%)
Gastric	4 (18.18%)
Mesothelioma	1 (4.55%)
Cytoreduction	
Complete (CC-0)	68 (80%)
Optimal (CC-1)	9 (10.6%)
Incomplete (CC-2)	7 (8.2%)
Non-resectable (CC-3)	1 (1.2%)
Type of surgery	
Primary tumor	61 (70.9%)
Persistence	2 (2.3%)
Recurrence	15 (17.4%)
Second look	8 (9.3%)
HIPEC	
Oxaliplatin	55 (64.7%)
Mytomicin C	14 (16.5%)
Taxol	11 (12.9%)
Carboplatin	2 (2.4%)
Carboplatin + taxol	1 (1.2%)
Cisplatin + Doxorubicin	1 (1.2%)
Oxaliplatin + Doxorubicin	1 (1.2%)
EPIC	
No	14 (16.3%)
Yes	72 (83.7%)

Data are presented as mean (range) for continuous measures; number (%) for categorical measures. PCI: Peritoneal cancer index; HIPEC: hyperthermic intraperitoneal chemotherapy; EPIC: early postoperative intraperitoneal chemotherapy.

**Table 2:** Postoperative outcomes.

	*n* = 86
Clavien–Dindo classification	
Grade I	40 (46.5%)
Grade II	20 (23.3%)
Grade IIIa	7 (8.1%)
Grade IIIb	9 (10.5%)
Grade IVa	6 (7%)
Grade IVb	2 (2.3%)
Grade V	2 (2.3%)
Clavien–Dindo categories	
No morbidity	40 (46.5%)
Minor morbidity	20 (23.3%)
Major morbidity	26 (27.9%)
In hospital stay	17 (4–63)
30-day mortality	2.3%
Reintervention	
No	72 (83.7%)
Yes	13 (15.1%)

Data are presented as number (%) for categorical measures and mean (range) for continuous measures.

**Table 3:** Colorectal overall survival according to PCI.

PCI	*n*	Median OS	12 Months OS	36 Months OS	120 Months OS
0	6	127 months	100%	55.6%	55.6%
1–9	12	80 months	91.7%	56.2%	37.5%
10–19	2	16 months	66.7%	33.3%	33.3%
≥20	6	29 months	66.7%	0%	0%

**Table 4:** Ovarian overall survival according to PCI.

PCI	*n*	Median OS	12 Months OS	36 Months OS	120 Months OS
0	10	20 months	90%	48%	16%
1–9	13	50 months	85.1%	61.9%	24.8%
10–19	8	44 months	87.5%	54.7%	18.2%
≥20	5	22 months	60%	30%	30%

**Table 5:** Colorectal overall survival according to CCS.

CCS	*n*	Median OS	12 Months OS	36 Months OS	120 Months OS
CC-0	21	80 months	94.7%	59.2%	47.5%
CC-1	4	29 months	75%	0%	0%
CC-2	5	16 months	60%	30%	30%
CC-3	0	-	-	-	-

**Table 6:** Ovarian overall survival according to CCS.

CCS	*n*	Median OS	12 Months OS	36 Months OS	120 Months OS
CC-0	32	50 months	84.7%	51.3%	23.1%
CC-1	3	44 months	100%	100%	0%
CC-2	1	8 months *	0% *	0% *	0% *
CC-3	1	120 months *	100% *	100% *	0% *

* Data with one case only.

## Data Availability

The data presented in this study are available on request from the corresponding author. The data are not publicly available due to privacy restrictions.
